# Are the impacts of food systems on climate change being reported by the media? An Australian media analysis

**DOI:** 10.1017/S1368980023000800

**Published:** 2023-08

**Authors:** Nicole Atkinson, Megan Ferguson, Cherie Russell, Katherine Cullerton

**Affiliations:** 1 School of Public Health, The University of Queensland, Brisbane 4072, Australia; 2 School of Exercise and Nutrition Sciences, Deakin University, Geelong, Australia

**Keywords:** Climate change, Global warming, Food systems, Agriculture, Media analysis, Advocacy

## Abstract

**Objective:**

Food systems are a major contributor to climate change, producing one-third of global greenhouse gas emissions. However, public knowledge of food systems’ contributions to climate change is low. One reason for low public awareness may be limited media coverage of the issue. To investigate this, we conducted a media analysis examining coverage of food systems and their contribution to climate change in Australian newspapers.

**Design:**

We analysed climate change articles from twelve Australian newspapers between 2011 and 2021, sourced from Factiva. We explored the volume and frequency of climate change articles that mentioned food systems and their contributions to climate change, as well as the level of focus on food systems.

**Setting:**

Australia.

**Participants:**

N/A.

**Results:**

Of the 2892 articles included, only 5 % mentioned the contributions of food systems to climate change, with the majority highlighting food production as the main contributor, followed by food consumption. Conversely, 8 % mentioned the impact of climate change on food systems.

**Conclusions:**

Though newspaper coverage of food systems’ effects on climate change is increasing, coverage of the issue remains limited. As newspapers play a key role in increasing public and political awareness of matters, the findings provide valuable insights for advocates wishing to increase engagement on the issue. Increased media coverage may raise public awareness and encourage action by policymakers. Collaboration between public health and environmental stakeholders to increase public knowledge of the relationship between food systems and climate change is recommended.

Climate change, caused by an increase in human-induced greenhouse gas (GHG) emissions in Earth’s atmosphere, is a growing threat to human health^(1)^. Impacts of the changing climate include extreme weather events, sea level rise, food insecurity and loss of biodiversity^(2)^. The *Special Report on Global Warming of 1·5°C* by The Intergovernmental Panel on Climate Change highlights the urgent action needed to reduce emissions and mitigate the catastrophic effects of climate change^(2)^.

Around one-third of global GHG emissions are caused by food systems^(3,4)^. Food systems encompass food production, processing, packaging, distribution, storage, retailing, consumption and waste^(3)^. Every component of this system produces GHG emissions^(3)^. Therefore, decreasing GHG emissions from both the supply side (production, processing, packaging, distribution and storage) and demand side of food systems (consumption and waste) is essential to attenuate the adverse impacts of climate change^(2)^. This requirement is becoming more urgent as food systems are becoming more industrialised and energy intensive^(3)^.

Globally, food production, including agriculture and fishing, and emissions from inputs such as fertilisers are the key contributors to food system emissions^(3–6)^. Production of livestock alone is responsible for 18 % of total global GHG emissions^(7)^. This figure is likely to rise with the increasing human population, as the demand for food (in particular meat and dairy) is expected to increase by 73 % and 58 %, respectively, by 2050^(8)^. Land-use changes, the next largest contributor to food system emissions^(3–6)^, involve the transformation of natural ecosystems to agricultural land, for example, deforestation for the expansion of pasture or crops^(9)^. The contribution of land-use changes to GHG emissions is highest in low- and middle-income countries^(3)^. Conversely, food packaging, processing, storage and waste disposal contribute to most emissions in high-income countries^(3)^.

Per capita, Australia is one of the largest emitters of GHGs in the world, yet has limited policies in place to address these emission levels^(10)^. Agriculture alone contributes to 13·5 % of Australia’s total emissions, the majority of which are due to livestock methane production^(11)^. However, this figure is likely to be an underestimate, as emissions from agricultural machinery, fertilisers, pesticides, food transportation and fuel used to generate electricity for food production are not included, nor are emissions from land clearing^(11)^. Despite these concerning figures and the many academic studies and global reports published on the impact of food systems on climate change^(3,4)^, public knowledge of the issue is low^(12–14)^. One study found that public awareness of the role the livestock industry plays as a contributor to climate change was limited compared to other sectors^(15)^. In fact, over twice the number of participants in the study identified direct transport (exhaust) emissions as a main contributor, despite the livestock sector contributing almost equally to global GHG emissions^(15)^.

The function of mass media coverage in policy agenda setting is complex. One element of this influence is the role that media coverage plays in shaping the public’s awareness and perception of an issue^(16)^. By selecting which issues and topics receive media attention, and how that topic is portrayed and framed, focus is drawn to particular issues as both important and salient^(16)^. Ostensibly, the agenda of news media largely sets the agenda of the public^(16)^. Given the influence of public opinion on governments and policymakers, media coverage is thus an important component of agenda setting in policymaking^(17,18)^. Conversely, low media coverage of or ‘indexing’ of an issue may limit issue salience with the public, signalling to policymakers that there is limited public appetite for policy change^(17)^.

While there has been an abundance of studies published on media coverage of climate change in general, there is minimal research on how the media cover the impact of food systems on climate change^(13)^. To date, media analyses of food systems and climate change have focused predominantly on the relationship between livestock, as part of food production (a single component of food systems), and emissions^(12,13,19–23)^. These studies universally found low volumes of coverage on the relationship between animal agriculture and climate change, with little responsibility placed on governments or the agriculture industry^(12,13,19–23)^.

Only one paper, based in the USA, has examined media coverage of food systems’ contributions to climate change, finding only 2·4 % of articles mentioned food systems’ contributions to climate change^(24)^. There is no research exploring Australian media coverage of food systems’ contributions to climate change broadly. To address this gap, we aimed to investigate media coverage of food systems and their contributions to climate change in popular Australian newspapers, including which components of food systems were mentioned as drivers of climate change.

## Methods

We undertook a media analysis of Australian newspaper coverage of food systems’ contributions to climate change. This research method was used to collect and analyse information about the topics and reporting priorities present in Australian media coverage^(25)^.

### Data collection

We searched the Factiva database^(26)^ (an international news database with a collection of sources from multiple disciplines) for online and print articles covering climate change in popular Australian broadsheet and tabloid newspapers^(27)^ between 11 August 2011 and 11 August 2021. This timeframe was chosen to capture the frequency and volume of articles published in the lead up to prominent climate change conferences and reports, specifically the release of the Intergovernmental Panel on Climate Change Sixth Assessment on the 6 August 2021^(2,4,28,29)^. This ten-year time period was chosen as public policy scholars recommend this as the minimum time to examine a full policy cycle and corresponding beliefs about policy issues^(17)^. Collection of data after the Intergovernmental Panel on Climate Change Sixth Assessment was not possible due to the time restrictions of our first author.

The following newspapers were searched for all available online and print climate change articles: *The Australian*, *Courier Mail*, *Daily Telegraph, The Age, Herald Sun, The Sydney Morning Herald, Hobart Mercury*, *The Advertiser, Northern Territory News, The Guardian*, *Canberra Times* and *The West Australian*. These newspapers were included as they have the highest readership of all Australian newspapers^(27)^. Both print and online newspaper versions were included where available from Factiva to accommodate for the rise in use of online news media^(30,31)^.

Newspapers were chosen for this study as they maintain a key role in shaping public understanding and perception of issues and can impact public policy^(24,32,33)^. Further, newspapers have been found to have a stronger influence on shaping public perceptions than television news^(32)^.

### Search strategy

A preliminary search of media articles on Factiva informed the search terms used in our study: *climate change OR global warming OR greenhouse gas OR climate emergency OR climate risk OR climate crisis*. Eligible articles were extracted from Factiva and screened using the inclusion/exclusion criteria (Table 1). Articles were included if at least 50 % of paragraphs related to climate change, as a stronger focus on climate change is more likely to have a lasting impression on the reader^(13)^. Consistent with the theory of news comprehension, where readers view the headline of an article first and then decide whether to keep reading or stop, we searched articles by title to ensure relevancy^(34)^.

**Table 1:** Inclusion and exclusion criteria

Inclusion criteria	Exclusion criteria
• Published in English. • News article, opinion, blog or commentary piece. • Published in an Australian newspaper. • Published between 11 August 2011 and 11 August 2021. • Minimum length of 100 words to ensure substantive coverage. • Containing at least 50 % of paragraphs relating to climate change.	• Published in a language other than English. • Letters, quizzes and magazine articles.

News articles and opinion, blog and commentary pieces were included, as they can all offer a unique perspective, and may provide opinion credibility with the intended audience^(21)^.

**Fig. 1:**
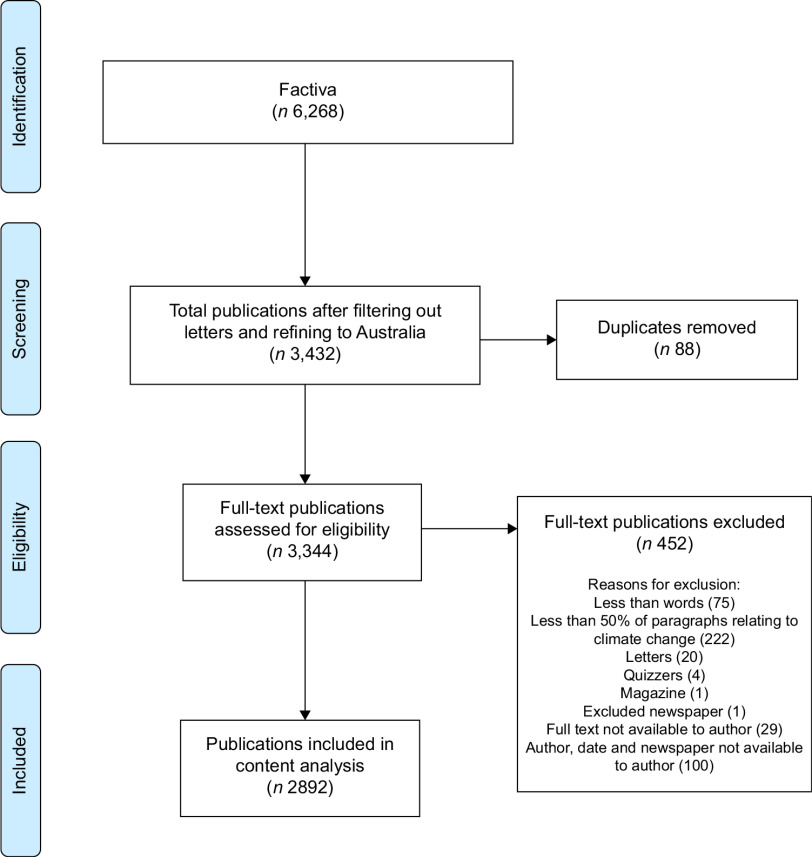
PRISMA flow chart outlining the search strategy used^(42)^

*Endnote X9* was used to collate articles, remove duplicates and screen articles. Replicate articles (the same article published by different newspapers) were included due to differing newspaper readership. Screening was completed by two reviewers. The primary reviewer (Author 1) screened 100 % of articles, while Author 4 screened 10 % of articles to ensure rigour. Reviewers revised all disagreements together at the end of the process. The data collection process is outlined in Fig. 1.

### Data analysis

We developed a coding framework, informed by previous research on climate change and media^(24)^. Data were extracted into a coding sheet in *Microsoft Excel (V.16.16.27)*. Extracted data included: journalist name, date of article, title of article, newspaper name, type of article, location in newspaper, mention of food systems, level of focus on food systems, whether food systems were mentioned as contributors to climate change (Yes or No) and component of food systems mentioned as a contributor to climate change (for example, food production)^(24)^. Multiple codes were attributed to each article. For instance, if food systems were mentioned, a secondary code was used to identify the level of focus on food systems (online Supplementary File 1). Articles that mentioned food systems were coded further if they mentioned food systems as a *contributor* to climate change, as opposed to a result of. These articles were then categorised, using definitions outlined in Table 2, into food production, food processing, food packaging and distribution, food storage, food preparation and food consumption as a contributor to climate change using the coding framework (online Supplementary File 1). Author 1 commenced coding using the coding framework with weekly discussions occurring with the team to clarify and refine codes. The weekly meetings were used as a means of reflexively improving the coding framework and analysis by provoking dialogue between researchers and identifying areas of coding that needed clarification. All researchers would read the relevant text and then discuss which code they believed was appropriate. Disagreements during this process were discussed between the team and this regular discussion allowed us to clearly identify how and why interpretations conflict and to improve the coding framework. To ensure consistency within coding decisions, Author 2 independently double coded 10 % of articles at the end of the coding process. Coder reliability was more than 95 % for coding food systems as a cause of climate change, and more than 70 % for coding the location of the newspaper story. In reviewing all disagreements between the two coders, consensus was reached on Author 1‘s original coding in each case. No codes were required to be changed as a result of this process. Content analysis and descriptive statistics were undertaken to examine the frequency and volume of climate change and food systems articles, and elements of food systems acknowledged as contributing to climate change.

**Table 2:** Definitions of each element of food systems

Element	Definition
Food production	Includes the use of land for agricultural purposes, crop production and management, livestock breeding and management, harvesting, manufacturing and food lost during this process^(43,44)^.
Food processing	Involves the processing of food after harvesting, such as handling, sorting, trimming and washing^(45)^.
Food packaging and distribution	Incorporates post-harvest activities such as food packaging and transportation from farms to communities^(43)^.
Food storage	Comprises of storing food for preparation and consumption, such as refrigeration^(45)^.
Food preparation	Includes preparing and cooking of food at home and in the community (including retail and dining)^(43)^.
Food consumption	Involves decision-making regarding food at the household level, food choices (such as eating meat and dairy) and food waste^(43)^.

## Results

### Level of climate change and food systems coverage over time

While there were many articles on climate change over the 10-year study period (*n* 2892), food systems coverage was low (*n* 380, 13 %) (Table 3). Articles either addressed the effect of climate change on food systems (*n* 224, 8 %) or the impact of food systems on climate change (*n* 144, 5 %). The remaining articles (*n* 12, 3 %) mentioned food systems, but did not address the interaction between climate change and food systems. Of the 144 articles that addressed the impact of food systems on climate change, only seventeen articles (< 1 %) contained more than three paragraphs focusing on the impact that food systems have on climate change.

**Table 3:** Coverage overview of climate change articles in 12 popular Australian newspapers from August 2011 to August 2021

	*n*	%
Climate change articles	2892	
Climate change articles that mentioned food systems	380	13·1
Climate change articles that mentioned the impact of climate change on food systems	224	7·7
Climate change articles that mentioned food systems as a contributor to climate change	144	5·0
‘Substantial’ focus (more than three paragraphs) on food systems as a contributor to climate change	17	0·6

**Fig. 2:**
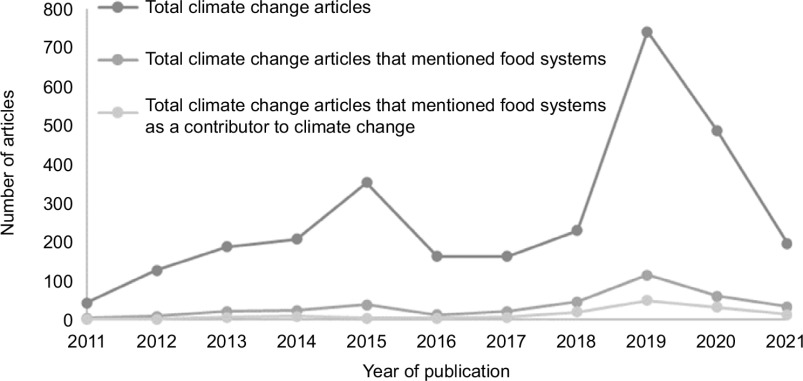
Total number of climate change articles in 12 popular Australian newspapers, August 2011 to August 2021. *Note: data collection started 11 August 2011 and finished 11 August 2021

As seen in Fig. 2, there was an increase in climate change articles over the 10-year study period, with a peak in 2019. A similar trend was observed for articles that mentioned food systems, as well as articles that recognised food systems as a contributor to climate change.

**Fig. 3:**
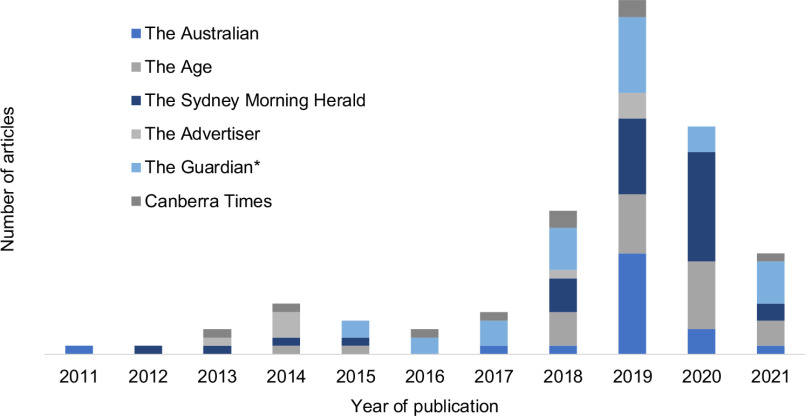
Total number of articles that acknowledged food systems as a contributor to climate change in the top six Australian newspapers, August 2011 to August 2021 * Note: The Guardian established Australian offices in 2014^(13)^. **Note: data collection started 11 August 2011 and finished 11 August 2021

The *Sydney Morning Herald* published the highest number of climate change articles (*n* 590, 20 %), followed by *The Guardian* (*n* 485, 17 %) and *The Australian* (*n* 449, 16 %). With regard to acknowledgement of food systems’ contributions to climate change, *The Sydney Morning Heral*d published the highest number of articles (*n* 32, 22 %), then The Guardian (*n* 29, 20 %) and The Age (*n* 24, 17 %) (Fig. 3). It is worth noting that The Guardian, a progressive online newspaper, only established Australian offices in 2014^(13)^. Even so, when comparing the number of articles produced by *The Guardian* and *The Sydney Morning Herald* between 2014 and 2021, the number of articles that mentioned food systems as a driver of climate change was similar (29 *v*. 30, respectively).

Most articles included in this analysis had a national focus (*n* 1923, 66 %) with a smaller proportion taking an international perspective (*n* 446, 15 %) and an even smaller number focusing on states within Australia. New South Wales was the focus of the most articles (*n* 168, 6 %), followed by Queensland (*n* 98, 3 %), Victoria (*n* 65, 2 %) and South Australia (*n* 62, 2 %). Similarly, of the 144 articles that mentioned the impact of food systems on climate change, the majority focused on Australia as a nation (*n* 110, 77 %). Of the remaining articles, 10 % (*n* 15) had an international focus, and 5 % (*n* 7) related to New South Wales.

### Content of climate change and food systems coverage

**Fig. 4:**
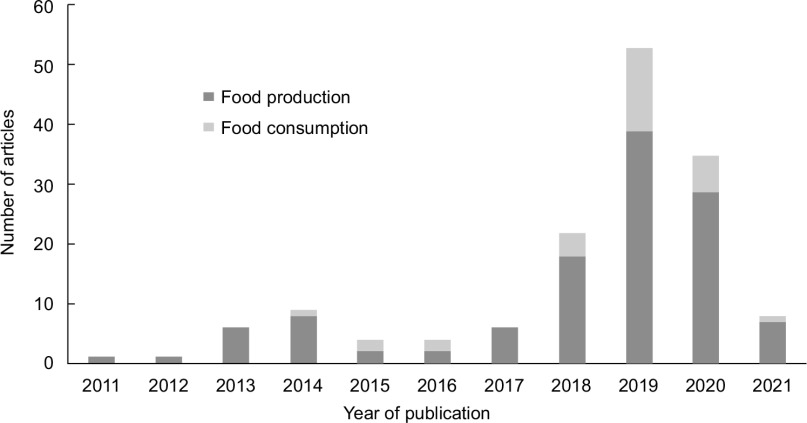
Elements of food systems acknowledged as contributors to climate change in 12 Australian newspapers, August 2011 to August 2021. *Note: data collection started 11 August 2011 and finished 11 August 2021

Most articles that acknowledged the role of food systems’ contributions to climate change (114 of the 144 articles, 79 %) highlighted the negative impact of food production, including production of crops and livestock, on climate change, while all others (*n* 30, 21 %) focused on food consumption (Fig. 4). Food processing, food packaging and distribution, food storage and food preparation were not acknowledged as drivers of climate change in any articles.

Among the articles that mentioned food production as a contributor to climate change, the majority referred to ‘meat and livestock (including animals used for meat and dairy)’ as the primary cause. Specifically, methane production from cattle was denoted as a major driver of climate change. On the other hand, dietary choices (such as choosing meat, livestock and dairy as food options), followed by food waste, were mentioned as the top contributors to climate change among articles that mentioned food consumption as a cause of climate change.

Finally, of the 224 articles that mentioned the impact of climate change on food systems, rather than the impact of food systems on climate change, almost 90 % (*n* 196) examined the negative impact that climate change had on agriculture, including farms, livestock and crops. The remaining proportion of articles (*n* 28) mentioned the positive impact that climate change had on food systems, such as increased carbon dioxide resulting in improved crop production and yield.

## Discussion

This study sought to investigate Australian media coverage of the relationship between food systems and climate change. Considering that one-third of GHG emissions are related to food systems^(3,4)^, our analysis demonstrates that coverage of the relationship between food systems and climate change is limited in popular Australian newspapers.

### Volume of coverage

Very few articles focused on food systems as a contributor to climate change (5 %), and even fewer articles focused substantially on this issue (< 1 %). To our knowledge, internationally, only one previous study has analysed newspaper coverage of food systems’ contributions to climate change. Findings from this study, based in the USA, indicated that between 2005 and 2008, 2·4 % of articles mentioned food systems’ contributions to climate change, with 0·4 % substantially focusing on the issue^(24)^. In comparison, our results suggest that Australian news coverage of this issue was slightly higher. However, the difference in results could be due to the recency of this study, as the USA paper analysed coverage between 2005 and 2008, and the awareness of the relationship between food systems and climate change has gained momentum in the past few years^(24)^.

Additionally, our study was the first to explore the proportion of articles that mentioned both the impact of food systems on climate change, and the effect of climate change on food systems. Neff, Chan and Smith excluded articles that focused solely on the effect of climate change on food systems^(24)^. Including these articles has provided important insights into media reporting priorities, with many more reporting on the impact of climate change on food systems rather than the impact of food systems on climate change.

### Content of coverage

Most articles that mentioned the impact of food systems on climate change highlighted food production, such as crop and livestock production, as a key contributor. This aligns with current evidence that the agriculture sector, as part of food production, is the main contributor to global food system emissions^(3–6)^. For articles that recognised the impact of food production on climate change, ‘meat and livestock (including animals used for meat and dairy)’ were referred to as the biggest contributors. This is supported by evidence, as the FAO predicts that the production of livestock alone is responsible for 18 % of total global GHG emissions^(7)^. In Australia, methane emissions from livestock are the leading contributor to agricultural emissions^(11)^. This was reflected in our analysis with methane production from cattle receiving a proportionately large amount of focus in the media.

Comparatively, we found that more articles mentioned the effect of climate change *on* food systems. The majority of these examined the negative impact that climate change had on agriculture, including farms, livestock and crops, for example the impact of drought on crop yields, and fires and floods killing livestock. These higher levels of reporting focused on the impact of climate change on agriculture may relate to the Australian public’s romanticised views about farming, partly due to the media, and discourses around the strength, resilience and trustworthiness of Australian farmers^(35–37)^. Indeed, research has demonstrated that Australians view agriculture to be very important to the future of the country^(35)^. Literature has shown that there is an appreciation of farmers, as well as strong public support for providing more assistance to farmers and the agriculture sector^(35,36)^. The remainder of articles mentioned the positive influence of climate change, particularly on crop production and yield. It is worth noting that the majority of these articles focusing on the positive aspects were written by an Australian conservative commentator who is a well-known climate change sceptic^(38)^.

### Interplay between food industry and government

As discussed, we found very low levels of media coverage on the role food systems play in climate change. The low amount of coverage could be explained in part by the reluctance of the Australian government to implement policies on this issue^(13)^. One reason for limited policy action is that powerful actors from the agriculture industry have lobbied successive governments to limit action on climate change^(13)^. Indeed, the powerful voices of climate sceptics continue to hold power within the Australian government^(38)^. Governments typically fail to act when faced with obstacles from powerful oppositions, putting greater responsibility on individuals for climate change mitigation^(23)^. The agriculture industry is very powerful within Australia as it is a large part of the nation’s economy^(12)^. The industry is a major employer in rural areas, and a major donor to conservative political parties^(12,39)^.

### Competition for media attention

There are many global issues that compete for media attention. Natural disasters, pandemics and other major events are often seen as more ‘newsworthy’ and take priority for news coverage. The competition for media attention is demonstrated by the findings of this study which indicated a dramatic increase in climate change articles published in 2019, partly explained by the Australian bushfires. The volume of articles decreased considerably in 2020 and 2021. This is likely due to the outbreak of COVID-19 internationally at the beginning of 2020 and the continued public interest in COVID-19.

Achieving greater media coverage of the impact of food systems on climate change will require climate and health advocates to raise public awareness of the issue and to take an active role in stimulating media coverage^(12)^. One study suggests that improved awareness of issues can be achieved if advocates work closely with journalists to make the story compelling to the public, supply journalists with fact sheets or visual aids, and appeal to the ethical values of the journalist^(40)^. This is essential to place food systems’ emissions on the national agenda, with the hope that policymakers respond to increased public interest by implementing mitigation policies that address both the supply and demand side of food systems^(41)^.

### Strengths and limitations

To our knowledge, this is the first Australian study to analyse media coverage of the relationship between food systems and climate change. This analysis used a much larger sample of newspapers over a longer time period (10 years) compared to other media analyses, with most other studies using one to four newspapers^(12,13,19–24)^. Additionally, this study included articles from newspapers across all states and territories of Australia, which captures articles aimed at a wide variety of readers. Furthermore, both online and print articles were included. Online articles have not been included in prior analyses^(19,22–24)^. Finally, the total dataset were coded, whereas similar media analyses only coded a sample of articles^(20,24)^.

The primary limitation of this study is that the headline search may have missed relevant climate change articles. Furthermore, double coding occurred at the end of the analysis and a statistical method for determining intercoder reliability was not used. However, we attempted to mitigate this limitation by ensuring frequent meetings with the research team throughout the coding process, particularly at the start, to discuss coding decisions and to improve the coding framework based on these discussions. Finally, media coverage may differ across different channels, such as television and social media. This study did not capture other news mediums or popular online news websites.

## Conclusions

Food systems are a key driver of climate change globally, which is resulting in adverse health and environmental impacts. By conducting a media analysis, we found that the coverage of contributions of food systems to climate change were low in Australian newspapers. While coverage of the issue is improving, without further meaningful coverage it is doubtful that the issue will enter mainstream public discourse, which may impact policy actions to attenuate climate change in Australia. Given that Australia is one of the largest emitters of GHG in the world per capita, we encourage Australian newspapers to increase their coverage of the impact that food systems have on climate change. Future research should explore the framing of the media coverage of food systems and climate change. Doing so may provide insight into whether there are opportunities for improved framing of the issue to ensure this topic resonates with the general public and inspires change.

## Supplementary material

For supplementary material accompanying this paper visit https://doi.org/10.1017/S1368980023000800. click here to view supplementary material
