# Supplementary material for: Are the impacts of food systems on climate change being reported by the media? An Australian media analysis
Source: Public Health Nutr. 2023 Apr 27;26(8):1706–14. doi: 10.1017/S1368980023000800 (PMC10410392; doi:10.1017/S1368980023000800)
Supplement: Supplementary file 1 [file S1368980023000800sup001.docx]

**Supplementary Material**

Supplement 1. Coding framework

| Location | Level of focus on food systems | Element of food systems acknowledged as a contributor to climate change – primary code | Element of food systems acknowledged as a contributor to climate change – secondary code |
| --- | --- | --- | --- |
| National | Minimal (less than one paragraph) | Food production | a. Meat and livestock (including animals used for meat and dairy)  b. Grains  c. Land use  d. Pesticides  e. Fertilisers  f. Vehicle pollution  g. Food loss |
| Victoria | Moderate (one to three paragraphs) | Food processing | - |
| New South Wales | Substantial (more than three paragraphs) | Food packaging and distribution | a. Packaging waste  b. Food transportation |
| Western Australia |  | Food storage | a. Refrigeration |
| Queensland |  | Food preparation | - |
| South Australia |  | Food consumption | a. Food waste  b. Dietary choices (such as choosing meat, livestock and dairy as food options) |
| Northern Territory |  |  |  |
| Tasmania |  |  |  |
| Australian Capital Territory |  |  |  |
| International |  |  |  |
